# Trajectory prediction model of diabetes distress in adults with type 2 diabetes mellitus: a 12-month prospective longitudinal study

**DOI:** 10.3389/fpsyg.2026.1510444

**Published:** 2026-02-26

**Authors:** Yu-Yun Zhang, Wei Li, Qing-Yan Wang, Fang Zhao, Qun Wang, Yu Sheng

**Affiliations:** 1 School of Nursing, Chinese Academy of Medical Sciences & Peking Union Medical College, Beijing, China; 2 School of Nursing, Kunming Medical University, Kunming, Yunnan, China; 3 Department of Endocrinology, Peking Union Medical College Hospital, Beijing, China; 4 School of Nursing, Xuzhou Medical University, Xuzhou, Jiangsu, China; 5 Department of Endocrinology, China-Japan Friendship Hospital, Beijing, China; 6 Endocrinology and Metabolism Department, Peking University Third Hospital, Beijing, China

**Keywords:** diabetes distress, growth mixture modeling, longitudinal study, trajectory, type 2 diabetes mellitus

## Abstract

**Introduction:**

Clarifying heterogeneous diabetes distress (DD) trajectories and their predictors from a dynamic perspective is crucial. We aimed to develop a trajectory prediction model for dynamic DD.

**Methods:**

This prospective longitudinal study included 443 adults with type 2 diabetes mellitus who completed the demographics and diabetes characteristics questionnaire, scales measuring lifestyles and psychological factors (at baseline), and the Chinese version of the Diabetes Distress Scale (at baseline and at 3-, 6-, 9-, and 12-month follow-ups). After identifying the factors associated with DD, growth mixture modeling was used to determine latent longitudinal DD trajectory classes and develop a trajectory prediction model.

**Results:**

Five DD trajectories were identified: persistently low DD (65.01%), persistently moderate DD (25.28%), persistently high DD (3.61%), decreasing DD (3.16%), and increasing DD (2.94%). Using the persistently low DD group as the reference, people with no religious belief (*B* = −24.932, *p* < 0.001), longer diabetes duration (*B* = 0.042, *p* = 0.037), worse self-management behaviors (*B* = −0.032, *p* = 0.009), and lower self-efficacy (*B* = −0.287, *p* = 0.007) tended to have a persistently moderate DD trajectory. Insomnia severity (*B* = 0.232, *p* = 0.008) and type D personality (*B* = 2.783, *p* = 0.002) were significant positive predictors of persistently high DD trajectory. Those with higher HbA1c levels (*B* = 0.728, *p* = 0.003) and lower self-efficacy (*B* = −0.858, *p* = 0.044) were more likely to belong to the decreasing DD trajectory class. Self-management behaviors (*B* = −0.127, *p* = 0.012) were negatively associated with belonging to the increasing DD trajectory class.

**Conclusion:**

Demographics, diabetes characteristics, lifestyles, and psychosocial factors can predict dynamic heterogeneous trajectories of DD. The trajectory prediction model will enable healthcare professionals to anticipate DD trajectories and conduct targeted interventions [Trial registration: ChiCTR2100047071].

## 1 Introduction

Diabetes is a group of chronic metabolic diseases characterized by defective insulin secretion and functionality, resulting in hyperglycemia (International Diabetes Federation, 2021). In 2021, 537 million adults aged 20–79 years had diabetes, accounting for 10.50% of the adult population, and approximately 144 million adults in China had diabetes, with a prevalence of 13.00% (International Diabetes Federation, 2021). Type 2 diabetes mellitus (T2DM), accounting for 96% of all diabetes cases, is expected to increase rapidly over the next 25 years (Ong et al., 2023).

T2DM not only damages organs and leads to complications, disability, and death but also causes mental health problems (Ali et al., 2013; Gordon, 2022). Diabetes distress (DD) is a diabetes-specific psychological problem, signifying a set of negative emotional responses and affective experiences, such as frustration, shame, worry, anger, fear, and guilt, due to diabetes-related demands and challenges (Polonsky et al., 2022). The prevalence of moderate and high DD in adults with T2DM was approximately 36% globally and 53% in China (García-Lara et al., 2022; Tang et al., 2022). In adults with T2DM, DD directly or indirectly reduces patient treatment adherence and self-management ability, leading to poor glycemic control and increased risk of complications and death (Kampling et al., 2022). In several American and British longitudinal studies involving 194–1,651 participants, DD was significantly associated with increased glycated hemoglobin (HbA1c) levels over 2 years from baseline (Fisher et al., 2010; Ismail et al., 2017; Marengo et al., 2021). Large-scale prospective cohort studies, conducted over an average of 7 years in Japan and 9 years in the United States, showed that patients with DD had significantly higher rates of complications and all-cause mortality (HR = 1.67–3.30) than those without DD (Hayashino et al., 2020; Huang et al., 2021). Thus, monitoring and managing DD in clinical nursing routines have been recommended (Skinner et al., 2020).

Studies focused on monitoring DD found that DD dynamically fluctuates over time and shows diverse trajectories owing to individual differences (Hendrieckx et al., 2019). Adults with T2DM in Canada were identified to exhibit five DD trajectories (persistently low, persistently low but at-risk, decreasing moderate, increasing moderate, and persistently severe) over 3 years through latent class growth modeling (Lipscombe et al., 2015). Moreover, DD changes over a certain period mediated the effect of the initial DD on 18-month HbA1c (Qian et al., 2023). Therefore, controlling and reducing DD is an important step in T2DM management. Studies proved that effective self-management education, peer support, and psychological interventions can significantly and rapidly reduce DD, with the intervention effect persisting for an extended period (Abbas et al., 2023; Fisher et al., 2020; Tang et al., 2022). Patients with persistently severe, moderate, or increasing moderate DD were more likely to benefit from these interventions (Lipscombe et al., 2015). Thus, predictive identification of these target patients can improve the effectiveness of interventions for the long-term management of DD and T2DM (Sachar et al., 2020). In summary, it is important to clarify heterogeneous DD trajectories and their predictors from a dynamic perspective. The trajectory prediction model uses baseline characteristics to identify patient subgroups with distinct long-term dynamic change patterns, enabling early recognition of potential high-risk individuals for targeted and personalized interventions (Kwon et al., 2021).

Characteristics of individuals with T2DM, including demographics (e.g., sex, age, educational level, employment status), T2DM characteristics (e.g., disease duration, glycemia, complications and comorbidities, treatment regimens), lifestyle factors (e.g., self-management behaviors, bad living habits), and psychosocial factors (e.g., personality, self-efficacy, coping styles, hope, social support), are associated with DD (Alzubaidi et al., 2022; Kamrul-Hasan et al., 2022; Kintzoglanakis et al., 2022; Lee et al., 2023; Liew and Vanoh, 2022; McCoy and Theeke, 2019; Seddigh and Tang, 2022). However, their predictive roles on distinct DD trajectories were rarely analyzed, making growth mixture modeling (GMM) valuable for identifying heterogeneous patterns. GMM offers unique advantages over traditional models by capturing individual variations and identifying latent unobserved trajectory classes rather than assuming population homogeneity, enabling the adoption of personalized interventions tailored to individual patterns (Kwon et al., 2021).

Thus, this study aimed to construct a trajectory prediction model using GMM for early identification of different DD trajectories in adults with T2DM.

This study tested two hypotheses: (1) repeated measures of DD over 1 year can identify 3–5 latent DD trajectory classes, and (2) baseline demographics, T2DM characteristics, lifestyles, and psychosocial factors can predict different trajectories.

## 2 Materials and methods

### 2.1 Study design and population

We conducted this longitudinal study (Trial registration: chictr.org.cn, ChiCTR2100047071) in the outpatient settings of the department of endocrinology across three class A tertiary comprehensive hospitals in Beijing, China. Recruitment was conducted from 7 June 2021 to 9 March 2022 using convenience sampling, and follow-ups were concluded on 9 March 2023. The sample size required for a maximum of six classes with high class separation in GMM was determined to be 300 (Kim, 2012).

We recruited adults based on the following inclusion criteria: diagnosed with T2DM by endocrinologists according to the World Health Organization’s 1999 Diagnosis and Classification of Diabetes Mellitus, age ≥18 years, conscious, could understand and communicate in written or spoken Chinese, signed informed consent forms, provided effective contact information, voluntarily participated, and self-reported the ability to attend follow-up visits on time.

The exclusion criteria comprised severe cognitive impairment; inability to cooperate, express clearly, or act independently; acute or severe complications (e.g., diabetic ketoacidosis, hypoglycemic episode, dialysis); serious comorbidities (e.g., severe or acute cardiac, cerebral, pulmonary, hepatic, and renal diseases); severe or acute infections; tumors; immune system diseases or hematologic diseases; severe mental disorders (schizophrenia, schizoaffective disorder, paranoid psychosis, bipolar affective disorder, mental disorders due to epilepsy, intellectual disability with mental disorders), or long-term use of antipsychotic drugs; and missing > 10% of all investigated items.

### 2.2 Measurements

In line with the requirements of GMM and previous studies focusing on emotional concerns (Baines et al., 2002; Wang and Bi, 2018), we measured DD at baseline (T0) and at 3- (T1), 6- (T2), 9- (T3), and 12-month (T4) intervals using the Chinese version of the Diabetes Distress Scale (Chinese Diabetes Society, 2021), which evaluates the severity and sources of DD (Fisher et al., 2014; Zhang et al., 2022). The 6-point Likert scale contains 17 items, scored from ‘1 (not a problem)’ to ‘6 (a very serious problem)’, covering four dimensions: emotional burden, physician distress, regimen distress, and interpersonal distress. The average score of <2 indicates little or no DD, ≥2 but <3 indicates moderate DD, and ≥3 indicates high DD (Fisher et al., 2012). The C-DDS17 met the required configural, metric, scalar, and error variance invariance of the longitudinal measurements (root mean square error of approximation [RMSEA] < 0.08, standardized root mean square residual [SRMR] < 0.08; Supplementary Table A1).

We formulated a questionnaire collecting patient demographics (patient-reported age, sex, ethnic group, religious belief, and marital, educational, and employment status), T2DM characteristics (patient-reported diabetes duration, number of comorbidities and complications, family history, treatment, and payment methods for diabetes treatment and care costs; researcher-calculated body mass index [weight in kilograms divided by the square of height in meters]; and medical record of HbA1c), and T2DM-related lifestyle factors (patient-reported smoking and drinking status, and scale-measured self-management behaviors and insomnia severity).

Self-management behaviors were assessed using the Summary of Diabetes Self-Care Activities scale (Toobert et al., 2000; Wan et al., 2008), which consists of 11 items and 6 dimensions (general diet, specific diet, exercise, blood glucose testing, foot care, and prescribed medication). For each item, the number of days a patient performed the self-care activity in the past week is recorded as 0–7, and the fourth item is scored in reverse. The total score is 0–77 points, reflecting the wellness of self-management behaviors. The Cronbach’s alpha coefficient was 0.697.

The Insomnia Severity Index (ISI; Bastien et al., 2001) features one dimension with seven items assessing the subjective symptoms and physical and mental effects of insomnia in the past 2 weeks. Items 1–6 rate from “0 (none)” to “4 (extremely severe);” item 7 rates from “0 (very satisfied)” to “4 (very dissatisfied).” The total score is 0–28 points, with a higher score reflecting more severe insomnia. The Cronbach’s alpha coefficient was 0.894.

Regarding psychosocial factors, personality was determined using the Type D Personality Scale-14 (Bai et al., 2007; Denollet, 2005), measuring negative affectivity (NA) and social inhibition (SI) personality traits. Scores range from ‘0 (false)’ to ‘4 (true)’, and items 1 and 3 are reversely scored. The total score for each subscale is 0–28 points; NA ≥ 10 and SI ≥ 10 indicate type D personality, and otherwise indicate non-type D personality. The Cronbach’s alpha coefficient was 0.893.

Self-efficacy in diabetes management was assessed using the Self-Efficacy for Managing Chronic Disease Six-Item Scale (Lorig et al., 1996; Wang L. et al., 2021), covering symptom and disease management. Each item scores from “1 (not confident at all)” to “10 (absolutely confident).” The average score reflects the overall self-efficacy level. The Cronbach’s alpha coefficient was 0.925.

We evaluated participants’ coping styles using the 20-item Simplified Coping Style Questionnaire, divided into positive and negative coping styles (Chen et al., 2022). Scores range from “0 (never)” to “3 (always).” A higher mean score indicates more frequent use of the coping styles during setbacks. The Cronbach’s alpha coefficient was 0.839.

We used the 12-item Herth Hope Index to assess hope (Herth, 1992; Wu C. et al., 2021). Items are rated from “1 (strongly disagree)” to “4 (strongly agree).” The scale consists of three subscales: inner sense of temporality and future, inner positive readiness and expectancy, and interconnectedness with self and others. A higher total score reflects a higher level of hope. The Cronbach’s alpha coefficient was 0.872.

We used the 10-item Social Support Rating Scale (Wu X. et al., 2021) to assess participants’ social support, which includes 10 items divided into objective support, subjective support, and social support availability. Ordinal numbers represent scores of items 1–4 and 8–10; the score of item 5 is the sum of sub-items 5A–5E scoring from “1 (none)” to “4 (full support);” items 6 and 7 are scored by the number of support sources. The total score is 12–66, representing the social support level. The Cronbach’s alpha coefficient was 0.731.

### 2.3 Statistical analyses

Participants with >10% missing data at a follow-up point were considered not to have completed the corresponding follow-up, and participants who completed at least two measurements were included in the final analysis.

First, for all participants included in the final analysis, we used SPSS 27 (IBM, Armonk, NY, United States) to perform expectation maximization to fill in missing values at baseline, including duration of T2DM, HbA1c, BMI, and the score of the social support rating scale, and we used multiple imputation to fill in missing DD values in the follow-up data. Next, the normality of continuous variables was tested by the Shapiro–Wilk test. Then, we used mean and standard deviation (mean [SD]) to describe normally distributed continuous variables, median and interquartile range (median [IQR]) for non-normally distributed continuous variables, and frequencies and percentages for binary variables. Furthermore, we conducted Spearman’s rank correlation to analyze the correlations between DD at five time points (T0–T4) and other variables measured at baseline.

Subsequently, we used Mplus 8.3 (Muthén & Muthén, Los Angeles, CA, USA) to analyze the trajectory prediction model of DD. We used C-DDS17 scores as latent trajectory class indicator variables, and DD correlators as potential predictors of different DD trajectory classes. The models were fitted through the robust maximum likelihood estimation due to the non-normal distribution of DD. The analysis steps were as follows:

(1) We conducted linear, quadratic (T0–T4 were set as 0–4), and time-score-free-estimated (T0–T1 were set as 0–1, and T2–T4 were free estimated) latent growth curve modeling (LGCM) of DD. Then, we selected the best-fitting model following the criteria that the ratio of chi-square to degrees of freedom (*χ*^2^/*df*) > 1 and <3, comparative fit index (CFI) > 0.90, Tucker–Lewis index (TLI) > 0.90, RMSEA <0.08, and SRMR <0.08. Smaller Akaike information criterion (AIC), Bayesian information criterion (BIC), and sample size adjusted BIC (aBIC) indicated a better-fitting model, and the lowest aBIC was more important than AIC and BIC in choosing a better model.
(2) The best-fitting LGCM guided the latent class growth modeling (LCGM) for 1–6 latent classes with all growth factor variances and covariances equal to 0; GMM for 1–6 latent classes with equal growth factor variances and covariances across classes; and GMM with freely estimated growth factor variances and covariances across classes. For LCGM and GMM, we used the R3STEP method, which automatically applied three steps to evaluate relationships between latent classes and predictors: (a) estimated the model using DD without additional variables; (b) created the most likely latent classes based on posterior probabilities obtained in the first step; (c) regressed latent classes on predictors with multinomial logistic regression while adjusting for classification uncertainty in the second step (Kwon et al., 2021).
(3) Finally, we selected the optimal model following the criteria wherein smaller values of information criteria indicated a better-fitting model, and aBIC was more important than AIC and BIC; an entropy value of ≥0.60 suggested at least 80% correct class assignment; *p*-values of the Lo–Mendell–Rubin likelihood ratio test (*p*LMR) and bootstrapped likelihood ratio test (*p*BLRT) of <0.05 indicated significantly better fitting of the k-class model than that of the k-1 class model; results were interpretable. In the optimal model, *p* < 0.05 of a variable indicated its role as a predictor of the latent trajectory class. The regression coefficient size and direction determined the predictive effect size and prediction direction of a predictor, respectively.

## 3 Results

### 3.1 Population characteristics at baseline and DD at five time points

Among the 936 participants who met the inclusion criteria and completed baseline measurement, only 443 individuals (47.33%) who volunteered for follow-ups and completed at least one follow-up measurement were included. The follow-up rates of the 443 participants at 3 (T1), 6 (T2), 9 (T3), and 12 (T4) months were 83.75, 68.62, 55.98, and 52.82%, respectively. These rates met the follow-up rate criterion (≥50%) for longitudinal studies (Fewtrell et al., 2008). Comparing baseline characteristics between participants and non-participants at each follow-up point using Mann–Whitney *U* test and chi-squared test revealed that baseline characteristics were generally comparable between the two groups, with only employment status (*χ*^2^ = 5.098, *p* = 0.024) and family history (*χ*^2^ = 4.965, *p* = 0.026) at T2, marital status (*χ*^2^ = 4.489, *p* = 0.034) at T3, and smoking status (*χ*^2^ = 7.978, *p* = 0.005) at T4 showing statistically significant differences (Supplementary Table A2).

Table 1 shows the baseline characteristics of the participants. The median (IQR) age, T2DM duration, and HbA1c level were 54.00 (41.00, 64.00) years, 5.00 (0.93, 12.00) years, and 6.90% (6.20, 8.20%), respectively. Among the participants, 54.85% (*n* = 243) were males, 95.49% (*n* = 423) were Han, and 97.07% (*n* = 430) were receiving medication or lifestyle treatment.

**Table 1:** Population characteristics at baseline and Spearman’s rank correlation coefficients with DD at five time points (*N* = 443).

Variables	Median (IQR)/Mean±SD/n (%)	Correlation coefficients between baseline variables and DD at five time points [*ρ* (*p*-value)]				
		T0	T1	T2	T3	T4
Age (year)	54.00 (41.00, 64.00)	−0.055 (0.244)	−0.073 (0.125)	−0.064 (0.179)	−0.080 (0.092)	−0.047 (0.327)
Sex		0.038 (0.429)	0.074 (0.122)	0.065 (0.172)	0.052 (0.275)	0.029 (0.546)
Male	243 (54.85)					
Female	200 (45.15)					
Ethnic group		0.011 (0.818)	0.078 (0.099)	0.025 (0.598)	0.028 (0.560)	0.082 (0.084)
Han	423 (95.49)					
Ethnic minorities	20 (4.51)					
Religious belief		0.073 (0.124)	0.071 (0.134)	0.086 (0.071)	0.096^*^ (0.044)	0.088 (0.065)
Have	24 (5.42)					
None	419 (94.58)					
Marital status		−0.057 (0.229)	−0.073 (0.127)	−0.033 (0.483)	−0.061 (0.203)	−0.058 (0.220)
Have a spouse (Married)	380 (85.78)					
No spouse (Unmarried/divorced/widowed)	63 (14.22)					
Educational status		0.021 (0.665)	0.034 (0.469)	0.036 (0.452)	0.016 (0.733)	0.021 (0.655)
Secondary school and below	129 (29.12)					
College and above	314 (70.88)					
Employment status		0.053 (0.263)	0.094^*^ (0.048)	0.079 (0.098)	0.073 (0.125)	0.052 (0.271)
Employed	239 (53.95)					
Unemployed	204 (46.05)					
Diabetes duration (year)	5.00 (0.93, 12.00)	0.090 (0.058)	0.059 (0.215)	0.096^*^ (0.044)	0.087 (0.068)	0.124^*^ (0.009)
Number of comorbidities and complications	3 (1, 4)	0.068 (0.150)	0.090 (0.057)	0.053 (0.268)	0.078 (0.101)	0.099^*^ (0.038)
Family history		−0.023 (0.625)	−0.035 (0.468)	−0.007 (0.888)	−0.036 (0.456)	−0.024 (0.611)
Yes	285 (64.33)					
No	158 (35.67)					
Treatment status		0.040	0.013	0.066	0.050	0.015
		(0.399)	(0.787)	(0.166)	(0.297)	(0.754)
Undergo treatment	430 (97.07)					
Not yet receive treatment	13 (2.93)					
Primary payment for medical expenses		−0.012 (0.802)	−0.057 (0.231)	−0.029 (0.539)	0.008 (0.865)	0.026 (0.589)
Reimbursement	393 (88.71)					
Self-payment	50 (11.29)					
Body mass index (kg/m^2^)	25.04 (22.58, 27.78)	0.037 (0.434)	0.045 (0.349)	0.007 (0.886)	0.045 (0.348)	0.036 (0.447)
HbA1c (%)	6.90 (6.20, 8.20)	0.196^**^ (<0.001)	0.118^*^ (0.013)	0.119^*^ (0.013)	0.140^**^ (0.003)	0.151^**^ (0.001)
Smoking		−0.059 (0.219)	−0.059 (0.216)	−0.111 (0.019)	−0.030 (0.523)	−0.017 (0.721)
Yes	95 (21.44)					
No	348 (78.56)					
Drinking		0.032 (0.505)	−0.017 (0.717)	0.010 (0.836)	0.013 (0.791)	0.035 (0.461)
Yes	169 (38.15)					
No	274 (61.85)					
Self-management behaviors	45.00 (34.00, 56.00)	−0.268^**^ (<0.001)	−0.211^**^ (<0.001)	−0.241^**^ (<0.001)	−0.248^**^ (<0.001)	−0.217^**^ (<0.001)
Insomnia severity	5.00 (2.00, 10.00)	0.373^**^ (<0.001)	0.415^**^ (<0.001)	0.351^**^ (<0.001)	0.334^**^ (<0.001)	0.356^**^ (<0.001)
Type D personality		0.312^**^ (<0.001)	0.275^**^ (<0.001)	0.281^**^ (<0.001)	0.325^**^ (<0.001)	0.281^**^ (<0.001)
Yes	123 (27.77)					
No	320 (72.23)					
Self-efficacy	7.67 (6.00, 8.83)	−0.461^**^ (<0.001)	−0.417^**^ (<0.001)	−0.443^**^ (<0.001)	−0.397^**^ (<0.001)	−0.409^**^ (<0.001)
Positive coping styles	2.17 (1.75, 2.58)	−0.166^**^ (<0.001)	−0.121^**^ (0.011)	−0.195^**^ (<0.001)	−0.200^**^ (<0.001)	−0.167^**^ (<0.001)
Negative coping styles	1.25 (0.88, 1.63)	0.095^**^ (0.045)	0.135^**^ (0.004)	0.152^**^ (0.001)	0.139^**^ (0.003)	0.155^**^ (0.001)
Hope	39.00 (35.00, 44.00)	−0.280^**^ (<0.001)	−0.275^**^ (<0.001)	−0.288^**^ (<0.001)	−0.321^**^ (<0.001)	−0.250^**^ (<0.001)
Social support	39.09 ± 8.04^†^	−0.227^**^ (<0.001)	−0.238^**^ (<0.001)	−0.227^**^ (<0.001)	−0.226^**^ (<0.001)	−0.241^**^ (<0.001)
DD (T0)	1.53 (1.18, 2.24)					
DD (T1)	1.65 (1.23, 2.29)					
DD (T2)	1.70 (1.24, 2.28)					
DD (T3)	1.82 (1.29, 2.51)					
DD (T4)	1.80 (1.26, 2.43)					

DD, Diabetes distress. ^†^The data follows a normal distribution. Statistical significance: ^*^p < 0.05, ^**^p < 0.01.

At baseline, the median score of C-DDS17 was 1.53 (1.18, 2.24). The prevalence of clinically significant DD (score≥2) was 33.18%, with moderate DD accounting for 24.83% (*n* = 110) and high DD accounting for 8.35% (*n* = 37) of all participants. During follow-up, the median DD scores ranged between 1.65 and 1.82.

### 3.2 Trajectory prediction model of DD

According to Spearman’s rank correlation results (Table 1), there were 14 DD correlators, including religious belief, employment status, diabetes duration, number of comorbidities and complications, HbA1c, smoking status, self-management behaviors, sleep quality, type D personality, self-efficacy, positive coping styles, negative coping styles, hope, and social support. All were potential predictors of DD trajectories.

The assignments of the predictors are listed in Supplementary Table A3, and the results of the LGCM, LCGM, and GMM are presented in Table 2. After considering the information criteria, classification accuracy, interpretability of results, and trajectory shapes, we concluded that the 5-class GMM with equivalent growth factor variance and covariance across classes was the most appropriate model.

**Table 2:** Fitting results of DD trajectory prediction model (*N* = 443).

LGCM	*χ* ^2^	*df*	*χ*^2^/*df*	CFI	TLI	AIC	BIC	aBIC	RMSEA	SRMR
Linear	17.349	10	1.735	0.992	0.992	4066.228	4107.164	4075.428	0.041	0.034
Quadratic	11.589	6	1.932	0.994	0.990	4066.504	4123.814	4079.385	0.046	0.032
Time scores freely estimated	11.245	7	1.606	0.995	0.993	4061.453	4114.669	4073.413	0.037	0.029

Profile	AIC	BIC	aBIC	*p*LMR	*p*BLRT	Entropy	Group size for each profile
LCGM (with variances and covariances of growth factors fixed at 0)							
1-class	5720.352	5761.288	5729.552	—	—	—	1.000
2-class	4635.160	4688.377	4647.121	0.001	<0.001	0.945	0.183/0.817
3-class	4207.269	4272.766	4221.989	0.010	<0.001	0.885	0.115/0.544/0.341
4-class	4009.386	4087.164	4026.867	<0.001	<0.001	0.896	0.129/0.490/0.343/0.038
5-class	3972.318	4062.377	3992.559	0.593	<0.001	0.804	0.300/0.370/0.190/0.038/0.102
6-class	3931.194	4033.533	3954.195	0.701	<0.001	0.819	0.079/0.194/0.025/0.302/0.363/0.036
GMM (with equivalent growth factor variances and covariances across classes)							
1-class	4061.453	4114.669	4073.413	—	—	—	1.000
2-class	3954.396	4019.893	3969.117	0.035	<0.001	0.905	0.099/0.901
3-class	3883.805	3961.583	3901.285	0.015	<0.001	0.886	0.700/0.059/0.241
4-class	3867.464	3957.523	3887.705	0.206	<0.001	0.889	0.029/0.059/0.237/0.675
5-class	3832.193	3934.532	3855.193	0.162	<0.001	0.899	0.036/0.650/0.253/0.032/0.029
6-class	3796.354	3910.974	3822.115	0.150	<0.001	0.891	0.066/0.036/0.221/0.036/0.056/0.585
GMM (with freely estimated variances and covariances of growth factors across classes)							
1-class	4061.453	4114.669	4073.413	—	—	—	1.000
2-class	3762.181	3852.239	3782.421	0.015	<0.001	0.732	0.589/0.411
3-class	3780.181	3907.081	3808.701	0.499	0.983	0.831	0.589/0.000/0.411
4-class	3798.181	3961.923	3834.981	0.498	0.984	0.866	0.000/0.589/0.000/0.411
5-class	3723.476	3924.061	3768.557	0.113	<0.001	0.792	0.548/0.000/0.359/0.093/0.000
6-class	3741.476	3978.903	3794.838	0.232	0.437	0.813	0.093/0.000/0.548/0.000/0.359/0.000

DD, Diabetes distress; LGCM, Latent growth curve model; LCGM, Latent class growth model; GMM, Growth mixture model. df, degree of freedom; CFI, comparative fit index; TLI, Tucker-Lewis index; AIC, Akaike information criterion; BIC, Bayesian information criterion; aBIC,sample size-adjusted BIC; RMSEA, root mean square error of approximation; SRMR, standardized root mean square residual; pLMR, *p*-value of Lo–Mendell–Rubin likelihood ratio test; pBLRT, *p*-value of bootstrapped likelihood ratio test.

**Figure 1:**
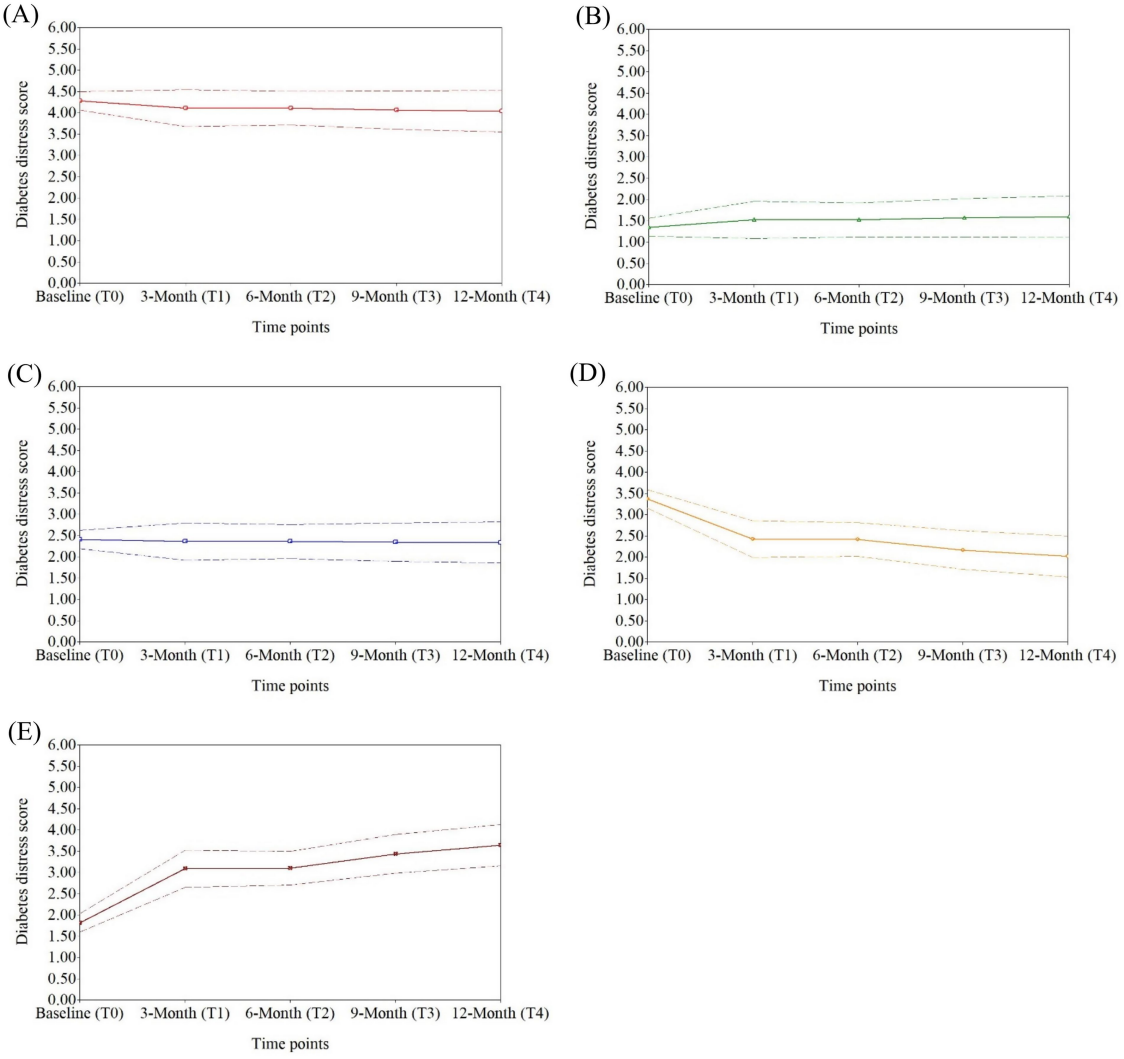
One-year diabetes distress trajectories of five latent classes represented in median and interquartile ranges. **(A)** Persistently high diabetes distress trajectory (Class 1); **(B)** Persistently low diabetes distress trajectory (Class 2); **(C)** Persistently moderate diabetes distress trajectory (Class 3); **(D)** Decreasing diabetes distress trajectory (Class 4); **(E)** Increasing diabetes distress trajectory (Class 5).

Based on the model, the DD trajectories were divided into five latent classes that were sufficiently clear to make a good distinction between different sub-populations (Figure 1). Class 2 was the largest group, comprising 288 (65.01%) participants, with a median score remaining in the low DD level, displaying a stable trajectory with a slight increase (persistently low DD trajectory). The second largest group was class 3 (persistently moderate DD trajectory), which consisted of 112 (25.28%) participants with persistently moderate levels of DD. Class 1 comprised 16 (3.61%) participants with consistently high DD scores above 3.5, showing a relatively stable pattern at the high DD level (persistently high DD trajectory). Class 4, comprising 14 (3.16%) participants, demonstrated a considerable reduction in DD scores from approximately 3.5 (high level) to 2.0 (moderate level) over time, thereby labeled as ‘decreasing DD trajectory’. Class 5 consisted of 13 (2.94%) participants with a median score that rapidly escalated from approximately 1.8 (low level) to over 3.0 (high level), denoted as ‘increasing DD trajectory’. Table 3 provides the estimated median values of DD per time point, along with the mean change rate and the corresponding *p*-value for each latent class.

**Table 3:** DD values at different time points and change rates of latent classes.

Latent classes (*n*, %)	DD values (Median)					Mean change rates and their *p*-values
	T0	T1	T2	T3	T4	
Class 1 (16, 3.61)	4.206	4.088	4.128	4.338	3.835	−0.172 (0.191)
Class 2 (288, 65.01)	1.294	1.412	1.412	1.529	1.472	0.172^**^ (<0.001)
Class 3 (112, 25.28)	2.412	2.235	2.235	2.353	2.307	−0.049 (0.447)
Class 4 (14, 3.16)	3.412	2.088	2.281	2.296	2.161	−0.946^*^ (0.016)
Class 5 (13, 2.93)	1.824	2.882	3.059	3.535	3.941	1.277^**^ (<0.001)

DD, Diabetes distress. Statistical significance: ^*^p < 0.05, ^**^p < 0.01.

Based on the multinomial logistic regressions from R3STEP using the persistently low DD trajectory (class 2) as the reference group, participants who had no religious belief (*B* = −24.932, *p* < 0.001), longer diabetes duration (*B* = 0.042, *p* = 0.037), worse self-management behaviors (*B* = −0.032, *p* = 0.009), and lower self-efficacy (*B* = −0.287, *p* = 0.007) tended to have the persistently moderate DD trajectory (class 3); insomnia severity (*B* = 0.232, *p* = 0.008) and type D personality (*B* = 2.783, *p* = 0.002) were the significant positive predictors of the persistently high DD trajectory (class 1); those with higher HbA1c levels (*B* = 0.728, *p* = 0.003) and lower self-efficacy (*B* = −0.858, *p* = 0.044) were more likely to belong to the decreasing DD trajectory (class 4); self-management behaviors (*B* = −0.127, *p* = 0.012) were negatively associated with belonging to the increasing DD trajectory (class 5; Table 4).

**Table 4:** Multinomial logistic regression analyses of predictors for latent classes of DD trajectories.

Factors	Regression coefficients (persistently low DD class [class 2] as the reference group)			
	*B*	*SE*	*t*	*p*
Persistently high DD (Class 1)				
Religious belief	3.199	1.701	1.880	0.060
Employment status	0.058	0.908	0.064	0.949
Diabetes duration	0.071	0.060	1.185	0.236
Number of comorbidities and complications	0.136	0.129	1.055	0.292
HbA1c	0.311	0.187	1.664	0.096
Smoking	−1.965	1.039	−1.892	0.059
Self-management behaviors	−0.035	0.035	−1.002	0.316
Insomnia severity	0.232	0.087	2.664	0.008^**^
Type D personality	2.783	0.913	3.047	0.002^**^
Self-efficacy	−0.168	0.227	−0.742	0.458
Positive coping styles	1.079	0.720	1.497	0.134
Negative coping styles	1.102	0.645	1.708	0.088
Hope	−0.059	0.047	−1.254	0.210
Social support	−0.021	0.048	−0.449	0.654
Persistently moderate DD (Class 3)				
Religious belief	−24.932	3.389	−7.356	<0.001^**^
Employment status	0.434	0.364	1.192	0.233
Diabetes duration	0.042	0.020	2.086	0.037^*^
Number of comorbidities and complications	0.079	0.073	1.079	0.281
HbA1c	0.102	0.075	1.355	0.175
Smoking	−0.136	0.391	−0.348	0.728
Self-management behaviors	−0.032	0.012	−2.601	0.009^**^
Insomnia severity	0.053	0.031	1.705	0.088
Type D personality	0.564	0.388	1.455	0.146
Self-efficacy	−0.287	0.106	−2.715	0.007^**^
Positive coping styles	0.651	0.432	1.507	0.132
Negative coping styles	−0.192	0.324	−0.593	0.553
Hope	−0.028	0.032	−0.849	0.396
Social support	−0.040	0.022	−1.857	0.063
Decreasing DD (Class 4)				
Religious belief	3.304	1.717	1.924	0.054
Employment status	1.396	1.568	0.890	0.373
Diabetes duration	−0.163	0.268	−0.607	0.544
Number of comorbidities and complications	0.392	0.223	1.755	0.079
HbA1c	0.728	0.248	2.937	0.003^**^
Smoking	−1.703	0.983	−1.733	0.083
Self-management behaviors	−0.080	0.049	−1.621	0.105
Insomnia severity	−0.081	0.091	−0.893	0.372
Type D personality	−0.181	0.952	−0.191	0.849
Self-efficacy	−0.858	0.427	−2.011	0.044^*^
Positive coping styles	−1.516	0.869	−1.744	0.081
Negative coping styles	0.848	0.825	1.028	0.304
Hope	0.099	0.126	0.784	0.433
Social support	0.069	0.059	1.160	0.246
Increasing DD (Class 5)				
Religious belief	5.246	3.389	1.548	0.122
Employment status	2.090	1.504	1.390	0.165
Diabetes duration	0.137	0.076	1.799	0.072
Number of comorbidities and complications	0.158	0.349	0.454	0.650
HbA1c	0.541	0.341	1.584	0.113
Smoking	−3.456	2.310	−1.496	0.135
Self-management behaviors	−0.127	0.051	−2.512	0.012^*^
Insomnia severity	0.211	0.171	1.231	0.218
Type D personality	−0.568	1.951	−0.291	0.771
Self-efficacy	0.032	0.488	0.066	0.948
Positive coping styles	2.003	1.254	1.598	0.110
Negative coping styles	2.834	1.453	1.950	0.051
Hope	0.000	0.112	−0.004	0.996
Social support	0.085	0.073	1.171	0.242

DD, Diabetes distress; B, Estimate of regression coefficient; SE, Standard error. Statistical significance: ^*^p < 0.05, ^**^p < 0.01.

## 4 Discussion

This study offers valuable insights into the trajectory patterns of DD over 1 year and the predictors of these trajectories among adults with T2DM. Using GMM, we identified five underlying trajectories labeled as persistently low DD, persistently moderate DD, persistently high DD, decreasing DD, and increasing DD. The proportions and trends observed in these groups highlight the variability in DD among individuals with T2DM. Furthermore, this study contributes to the literature by implicating the predictive effect of religious belief, diabetes duration, HbA1c, self-management behaviors, insomnia severity, type D personality, and self-efficacy on different DD trajectories.

The DD scores for the vast majority of participants (93.90%) remained relatively stable over time. This result was similar to that obtained by Fisher et al. (2022), who used the new DD measure scale and reported that approximately 80% of patients maintained stable levels of DD within half a year. Of note, a key contributor to the high stability rate is that the study was conducted in class A tertiary hospitals in China, which provide nationwide advanced medical care and comprehensive multidisciplinary supportive services for patients with T2DM. Such clinical environments with abundant resources ensure continuous access to standardized disease management and integrated psychological support, which in turn facilitate stable glycemic control and emotional well-being, thus corresponding to the stable DD trajectories observed in most participants. Therefore, caution is warranted when extrapolating the study findings to other contexts, as the favorable healthcare conditions, service structures, and cultural factors specific to the study setting may not be readily available in primary care facilities or resource-limited regions globally. In such settings, the predictive effects of the identified factors on DD trajectories may differ from those observed in our sample.

Among these individuals, 65.01% of the total sample maintained low levels of DD that closely paralleled a previously published study that found persistently low levels of DD in 61.15% of individuals during 4 years of follow-up (Lipscombe et al., 2015). This group seemed to exhibit no risk of developing moderate or high DD within a short time frame; however, considering the slight upward trend, monitoring once a year or every few years might still be necessary. While approximately two-thirds of the participants maintained low levels of DD, approximately a quarter experienced persistently moderate DD. This trajectory was related to no religious belief, longer diabetes duration, worse self-management behaviors, and lower self-efficacy when compared with the persistently low DD trajectory. The notable negative coefficient for religious belief (*B* = −24.932, *p* < 0.001) suggests individuals without religious belief may lack a potential psychological coping source for DD (Onyishi et al., 2022). Given the low proportion of participants with religious belief (5.42%), which aligns with the sociocultural context of China where diverse secular support systems play a dominant role, it is imperative to emphasize psychosocial support from families, communities, and healthcare providers as effective buffers against DD (Pan et al., 2024; Lee et al., 2023). The negative coefficients for self-efficacy (*B* = −0.287, *p* = 0.007) and self-management behaviors (*B* = −0.032, *p* = 0.009) reinforce that enhancing self-care skills and confidence in disease management may help reduce the risk of persistent moderate DD. For diabetes duration (*B* = 0.042, *p* = 0.037), each additional year of disease correlates with a small but meaningful increase in such risk, suggesting routine psychological assessment in long-term T2DM care.

Our findings also identified type D personality and insomnia severity as key factors for the persistently high DD trajectory class. These factors have also been identified in previous cross-sectional and longitudinal studies as important correlates or predictors of DD (Alzubaidi et al., 2022; Lee et al., 2023; McCoy and Theeke, 2019; Zhang et al., 2024). The robust positive regression coefficient for type D personality (*B* = 2.783, *p* = 0.002) indicates a clinically meaningful increased risk of individuals with this personality type being assigned to the persistently high DD trajectory. These patients tend to have long-term DD that impairs treatment adherence and glycemic control (Lee et al., 2023), which underscores the need to proactively screen T2DM outpatients for type D personality and implement targeted psychosocial interventions to break the cycle of chronic DD. Similarly, the positive coefficient for insomnia severity (*B* = 0.232, *p* = 0.008) implies that greater insomnia severity correlates with a rise in the risk of persistent high DD. As insomnia is modifiable, behavioral interventions or appropriate pharmacotherapy for sleep improvement could feasibly reduce chronic diabetes-related emotional burden (Jeon et al., 2023).

A small yet significant portion of participants exhibited extreme and dynamic changes in DD through rapidly increasing and decreasing trajectories. Adults with poorer self-management behaviors (*B* = −0.127, *p* = 0.012) may have a higher risk of rapid DD onset, even if they are not currently experiencing DD (increasing DD trajectory). Thus, for patients with T2DM, implementing early and proactive self-management education or interventions would be more beneficial in preventing the development of DD rather than waiting to address DD once it has manifested. In summary, adhering to standardized treatment and management guidelines might be important for controlling DD in T2DM.

Although cross-sectional analyses demonstrated consistently positive associations between HbA1c and DD and negative associations between self-efficacy and DD across all measurement points (baseline and 3-, 6-, 9-, and 12-month follow-ups), the study uniquely revealed a subtle dynamic trajectory class, unlike prior longitudinal studies that linked decreased HbA1c and improved self-efficacy to reduced DD (Qian et al., 2023; Wang et al., 2017). When using the persistently low DD trajectory class as the reference in GMM, baseline high HbA1c (*B* = 0.728, *p* = 0.003) and low self-efficacy (*B* = −0.858, *p* = 0.044) predicted categorization into the decreasing DD trajectory. Despite the significant decline, the DD level of the decreasing DD trajectory remained moderate throughout follow-up, indicating the persistent negative impact of hyperglycemia and low self-efficacy, and still aligning with the Spearman’s correlation analysis results to a certain extent.

The counterintuitive finding can be explained by the Stress and Adaptation Theory. According to this theory, high HbA1c and low self-efficacy are not merely static risk factors for DD, but rather key stressors that trigger physiological and psychological imbalances. Elevated HbA1c reflects inadequate glycemic control, while low self-efficacy stems from low confidence in disease management and poor mastery of self-care behaviors. Both of them contribute to heightened DD in cross-sectional observations, which capture only a single time point of stress-response imbalance. However, they also triggered robust adaptive responses under standard diabetes care including health education, medication guidance, psychological support, and self-management skill training. Specifically, patients with higher HbA1c tend to have greater crisis awareness, motivating them to engage more actively in treatment adherence and lifestyle modifications. Concurrently, those with lower self-efficacy benefit from structured education and psychological interventions, which help build their disease management competence and confidence. These ultimately lead to greater reductions in both glycemia and DD over time (Xin et al., 2024; Kintzoglanakis et al., 2022). The findings suggest that Stress and Adaptation Theory-guided interventions that optimizing glycemic control and self-efficacy can turn baseline stress-related disadvantages into opportunities to alleviate DD through successful stress adaptation.

To assess potential attrition bias and trajectory solution stability, we compared baseline characteristics between participants and non-participants at each follow-up point. Analyses revealed that baseline characteristics were generally comparable between the two groups with no statistically significant differences, except for the employment status and family history at T2, marital status at T3, and smoking status at T4 that showed statistically significant differences. These results suggest minimal attrition bias, as these variables were neither significantly correlated with DD nor identified as predictors of DD trajectories in analyses. Therefore, the limited discrepancies and negligible attrition bias was insufficient to undermine the relative stability of the trajectory prediction model. Nevertheless, unmeasured time-varying factors might still influence both participant retention and trajectory outcomes. Notably, this study only examined predictors at baseline without tracking their longitudinal changes, while the observed DD level changes likely reflects unmeasured shifts in life circumstances, psychological adaptation, and healthcare engagement under conventional medical care offered by Class A tertiary hospitals (Alzubaidi et al., 2022). These findings highlight the critical need to differentiate static cross-sectional associations from dynamic trajectory patterns, as well as and the importance of tracking changes in predictors alongside DD trajectories to elucidate mechanistic links. Causal inference is inherently constrained when relying solely on baseline data, because the influence of time-varying confounders that may both shape DD changes and correlate with baseline predictors cannot be rule out. Therefore, future studies incorporating repeated measurements of both predictors and potential confounders over time are essential to disentangle causal relationships and strengthen the validity of trajectory predictions.

Although GMM can technically accommodate varying class sizes, the modest sample sizes (*n* = 13–16) for the persistently high, decreasing, and increasing DD trajectories undermine the robustness of GMM and multinomial logistic regression estimates by potentially hindering the clear identification of meaningful latent classes, experiencing convergence issues, and producing unstable, less reliable growth factor parameters (Kwon et al., 2021). Additionally, while the overall entropy was high (0.899), indicating good classification quality at the model level, the assignment of individuals to smaller classes may be less precise, and the class solution could be sample-dependent. Consequently, the findings related to these three trajectories should be explicitly framed as exploratory and hypothesis-generating, with their identified predictors considered preliminary rather than definitive. The observed associations require confirmation in larger, more diverse cohorts to rule out chance findings and enhance generalizability. We specifically recommend future validation studies recruit samples with larger numbers of participants in extreme trajectory classes and include more heterogeneous populations to test the stability of the regression estimates and refine the prediction model. Besides, their clinical application should be deferred until validated in sufficiently powered studies.

The clinical value of identifying distinct DD trajectories lies in guiding the development of feasible, resource-efficient, and cost-effective care pattern for routine clinical practice through the RE-AIM (Reach, Effectiveness, Adoption, Implementation, Maintenance) implementation science lens. From the ‘Reach’ perspective, the model targets adult patients with T2DM, which is a large and growing population globally. From the ‘Effectiveness’ standpoint, the model ensures populations with distinct DD trajectory characteristics receive more targeted interventions tailored to their specific needs. Specifically, most patients (65.01%) with stable low DD need only annual screenings, freeing resources for higher-risk subgroups. Simultaneous, the model leveraged baseline predictors already collected in standard clinical assessments, eliminating extra data burden. The 28.89% with persistent moderate or high DD require urgent, focused care and intervention targeting identified predictors (e.g., severe insomnia, low self-efficacy) integrated with routine diabetes treatment, while the 2.94% with increasing DD need early self-management training to prevent DD from escalation, and the 3.16% with decreasing DD need confidence-boosting activities to sustain improvement. For ‘Adoption’ and ‘Implementation’, the model can be incorporated into existing Hospital Information Systems to facilitate seamless risk stratification without disrupting established workflows. ‘Maintenance’ may be reinforced by stable baseline predictors and the ability to refine the model with real-world data over time. The model also lays the foundation for developing stratified, trajectory-specific intervention strategies in future research, which can enhance the accuracy and cost-effectiveness of precision mental health care, thereby promoting the practical applicability and sustainability of the model beyond research and addressing existing resource constraints.

### 4.1 Strengths and limitations

We adopted a dynamic perspective to classify heterogeneous DD trajectories in T2DM and developed a prediction model using GMM with predictors. The model enabled early identification of high-risk individuals and laid a foundation for individualized early prevention of DD.

Despite the strengths, the findings should be interpreted and generalized cautiously given the following limitations. First, some of our groups included fewer than 50 individuals, which is acceptable for GMM because there is no strict minimum number of participants in each class. However, this may impact the generalizability and external validity of the study. Second, although the male-to-female ratio (1.22) and age distribution in this study were consistent with the epidemiological characteristics of populations with T2DM worldwide and in China, the participants had shorter median diabetes duration than those in previous studies (Ong et al., 2023; Zhang et al., 2020; Wang R. et al., 2021). Additionally, the participants had a median HbA1c level close to the glycemic control target, and nearly all patients undergoing treatment followed the Guidelines for the Prevention and Treatment of T2DM in China (2020 edition; Chinese Diabetes Society, 2021). Thus, selection bias may affect the generalization of the results to patients with longer T2DM duration, poorer glycemic control, or irregular treatments. Third, self-reported scales may be influenced by social expectation bias. Fourth, we did not include possible confounding factors such as time-varying factors (e.g., HbA1c and other interventions received during the follow-up period) in the analysis, which could have affected the results to a certain extent.

Thus, for future studies, we recommend conducting multi-regional, multi-centered, large-sample-sized prospective longitudinal studies with stratified sampling to enhance sample representativeness and improve the accuracy and stability of the trajectory prediction model. To improve data accuracy, incorporating biological indicators and electronic medical records is also necessary. Furthermore, we suggest controlling for confounding factors throughout the follow-up period to explore better-fitting trajectories and predictors.

## 5 Conclusion

This study aimed to identify various DD trajectories over 1 year, explore their predictors, and develop a prediction model using GMM among adults with T2DM. Five latent trajectories were identified: persistently low DD, persistently moderate DD, persistently high DD, decreasing DD, and increasing DD. Religious belief, diabetes duration, HbA1c, self-management behaviors, insomnia severity, type D personality, and self-efficacy were significant predictors of different DD trajectories. The study results offer a new perspective for future research on DD evolution and mental health interventions, though further verification is necessary owing to potential biases and the small sample size.

The prediction model enables the early identification of high-risk individuals, facilitating targeted support, and tailoring mental health interventions. Furthermore, the model can be integrated into the Hospital Information Systems (HIS), allowing healthcare providers to predict DD trends by inputting initial DD scores and their predictors. If a sufficient amount of data can be entered, analyzed, and tracked in the HIS, the model might be optimized in future clinical settings.

## Data Availability

The raw data supporting the conclusions of this article will be made available by the authors, without undue reservation.
