# Supplementary material for: Trajectory prediction model of diabetes distress in adults with type 2 diabetes mellitus: a 12-month prospective longitudinal study
Source: Front Psychol. 2026 Feb 26;17:1510444. doi: 10.3389/fpsyg.2026.1510444 (PMC12979389; doi:10.3389/fpsyg.2026.1510444)
Supplement: Supplementary file 1 [file supplementary_file_1.docx]

Supplementary Material

1. **Supplementary Data**

| **Table A1** Longitudinal measurement invariances of the Chinese version of Diabetes Distress Scale. | | | | | | |
| --- | --- | --- | --- | --- | --- | --- |
| Invariance | *χ*^2^ | *df* | *c* | CFI | RMSEA | SRMR |
| configural | 5635.745 | 3150 | 1.167 | 0.876 | 0.042 | 0.063 |
| metric | 5741.511 | 3202 | 1.174 | 0.874 | 0.042 | 0.065 |
| scalar | 5949.478 | 3270 | 1.169 | 0.867 | 0.043 | 0.070 |
| error variance | 6344.787 | 3338 | 1.208 | 0.850 | 0.045 | 0.072 |
| Note. *df*: Degree of freedom; *c*: Longitudinal measurement invariance coefficient; CFI: Comparative fix index; RMSEA: Root-mean-square error of approximation; SRMR: Standardized root mean square residual. | | | | | | |

| **Table A2** Comparison of baseline characteristics between participants and non-participants at each follow-up time point (*N*=443). | | | | | | | | | | | |
| --- | --- | --- | --- | --- | --- | --- | --- | --- | --- | --- | --- |
| Variables | T1 (371 participants, 72 non-participants) | |  | T2 (304 participants, 139 non-participants) | |  | T3 (248 participants, 195 non-participants) | |  | T4 (234 participants, 209 non-participants) | |
|  | *Z* / *χ*^2^ | *p* |  | *Z* / *χ*^2^ | *p* |  | *Z* / *χ*^2^ | *p* |  | *Z* / *χ*^2^ | *p* |
| Age (year) | -1.433^a^ | .152 |  | -1.858^a^ | .063 |  | .348^a^ | .728 |  | -.472^a^ | .637 |
| Sex | 1.360^b^ | .244 |  | .957^b^ | .328 |  | .341^b^ | .559 |  | .694^b^ | .405 |
| Ethnic group | .601^b^ | .438 |  | .018^b^ | .892 |  | .691^b^ | .406 |  | .514^b^ | .473 |
| Religious belief | .635^b^ | .426 |  | .479^b^ | .489 |  | .057^b^ | .811 |  | .018^b^ | .892 |
| Marital status | .682^b^ | .409 |  | 1.220^b^ | .269 |  | 4.489^b^ | .034^*^ |  | 3.356^b^ | .067 |
| Educational status | 1.307^b^ | .253 |  | 3.690^b^ | .055 |  | .218^b^ | .641 |  | .752^b^ | .386 |
| Employment status | .227^b^ | .634 |  | 5.098^b^ | .024^*^ |  | .119^b^ | .730 |  | .112^b^ | .737 |
| Diabetes duration (year) | -.528^a^ | .597 |  | -1.836^a^ | .066 |  | -.704^a^ | .482 |  | -.859^a^ | .390 |
| Number of comorbidities and complications | 1.225^a^ | .221 |  | .247^a^ | .805 |  | -.076^a^ | .940 |  | -.032^a^ | .974 |
| Family history | .389^b^ | .533 |  | 4.965^b^ | .026^*^ |  | 3.566^b^ | .059 |  | 2.196^b^ | .138 |
| Treatment status | .087^b^ | .768 |  | .123 | .725 |  | .168^b^ | .682 |  | .006^b^ | .940 |
| Primary payment for medical expenses | 1.368^b^ | .242 |  | 1.424^b^ | .233 |  | .828^b^ | .363 |  | 2.826^b^ | .093 |
| Body mass index (kg/m^2^) | -.196^a^ | .844 |  | 1.579^a^ | .114 |  | -.138^a^ | .891 |  |  |  |
| HbA1c (%) | -.373^a^ | .709 |  | -1.125^a^ | .261 |  | -.094^a^ | .925 |  | -.869^a^ | .385 |
| Smoking | .240^b^ | .625 |  | 1.678^b^ | .195 |  | 2.806^b^ | .094 |  | 7.978^b^ | .005^**^ |
| Drinking | .151 | .697 |  | 1.585^b^ | .208 |  | .014^b^ | .904 |  | .003^b^ | .958 |
| Self-management behaviors | .899^a^ | .369 |  | .563^a^ | .574 |  | .566^a^ | .571 |  | .558^a^ | .577 |
| Insomnia severity | 1.208^a^ | .227 |  | -.096^a^ | .924 |  | -1.359^a^ | .174 |  | -1.041^a^ | .298 |
| Type D personality | 2.968^b^ | .085 |  | .018^b^ | .892 |  | .373^b^ | .541 |  | .399^b^ | .528 |
| Self-efficacy | -1.236^a^ | .216 |  | -.770^a^ | .441 |  | 1.211^a^ | .226 |  | .286^a^ | .775 |
| Positive coping styles | .888^a^ | .375 |  | .646^a^ | .518 |  | .896^a^ | .370 |  | .814^a^ | .416 |
| Negative coping styles | .518^a^ | .604 |  | -1.129^a^ | .259 |  | -1.750^a^ | .080 |  | -1.481^a^ | .139 |
| Hope | -.312^a^ | .755 |  | .780^a^ | .435 |  | .755^a^ | .450 |  | .155^a^ | .877 |
| Social support | .450^a^ | .652 |  | .840^a^ | .401 |  | -.824^a^ | .410 |  | -.350^a^ | .727 |
| DD (T0) | 1.182^a^ | .237 |  | -.669^a^ | .504 |  | -1.172^a^ | .241 |  | -1.002^a^ | .316 |
| Note. ^a^ Z of the Mann-Whitney *U* test; ^b^ *χ*^2^ of the Chi-Squared Test. ^†^The data follows a normal distribution. Statistical significance: ^*^*p*<0.05；^**^*p*<0.01. | | | | | | | | | | | |

| **Table A3** Assignment of potential predictors of diabetes distress trajectory. | |
| --- | --- |
| Predictors | Assignment |
| Religion | 0 = None; 1 = Have |
| Employment status | 0 = Unemployed; 1 = Employed |
| Diabetes duration | Original value |
| Number of comorbidities and complications | Original value |
| HbA1c | Original value |
| Smoking | 0 = No; 1 = Yes |
| Self-management behaviours | Original value |
| Insomnia severity | Original value |
| Type D Personality | 0 = Non-type D personality; 1 = Type D personality |
| Self-efficacy | Original value |
| Positive coping styles | Original value |
| Negative coping styles | Original value |
| Hope | Original value |
| Social support | Original value |


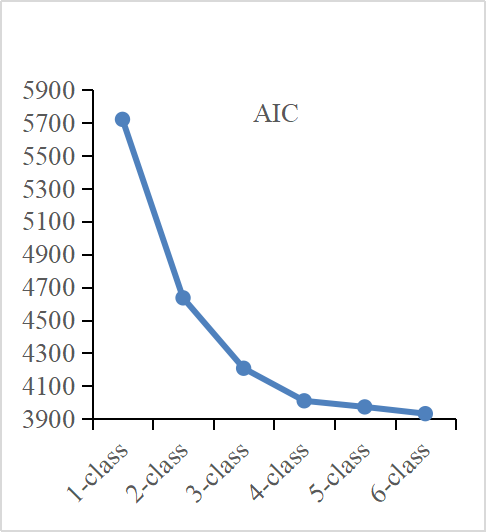

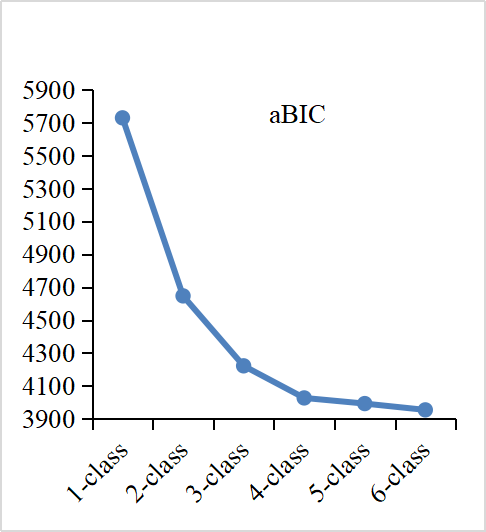

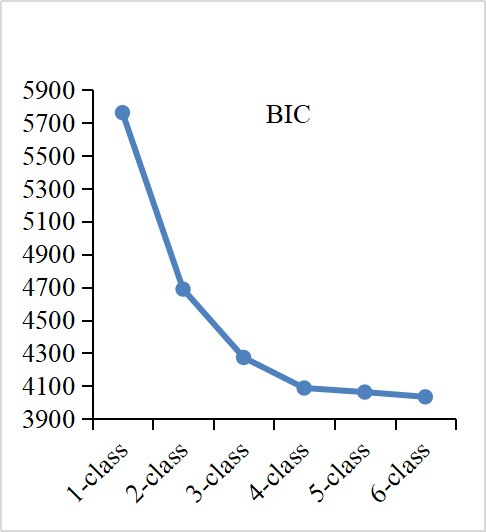


**Figure A1.** Scree plots of the AIC, BIC and aBIC of the latent class growth modelling

# Process and rationale for selecting the best model

Among the linear, quadratic, and time scores freely estimated LGCM, the model with freely estimated time scores showed the smallest AIC and aBIC, with 1<χ2/df<3, CFI and TLI>0.90, and RMSEA and SRMR<0.05, so it was confirmed as the most suitable model. Thus, we conducted time score freely estimated LCGM and GMM with 14 potential predictors using the R3STEP method.

In LCGM, AIC, BIC, and aBIC decreased when the number of latent classes increased, and the inflection point occurred in the 4-class model as shown in the scree plot (Figure A1). Meanwhile, pLMR, pBLRT and the entropy value suggested that the 4-class model fitted better than the 3-class model. Therefore, the 4-class model was the best-fitting LCGM.

In GMM with equivalent growth factor variance and covariance across classes, AIC, BIC and aBIC decreased when the number of latent classes increased, and pBLRT<0.001 suggested that k-class models fitted better than k-1-class models. Considering that the entropy of the 5-class model was higher than that of the 3-, 4-, and 6-class models, the 5-class model was the best-fitting GMM with equivalent growth factor variance and covariance across classes.

As for GMM with freely estimated variances and covariances of growth factors across classes, all models have a group with 0 participants except for the 2-class model, so only the 2-class model is acceptable.

After considering the information criteria, classification accuracy, interpretability of results, and trajectory shapes, we concluded that the 5-class GMM with equivalent growth factor variance and covariance across classes was the most appropriate model.
